# Hydrogen-Bond Scalar Couplings as Covalency-Sensitive NMR Fingerprints of Amorphous Ice

**DOI:** 10.1021/acs.jpcb.6c03372

**Published:** 2026-07-24

**Authors:** Hossam Elgabarty, Thomas D. Kühne

**Affiliations:** † Department of Chemistry, University of Paderborn, Warburger Str. 100, D-33098 Paderborn, Germany; ‡ 682670 Center for Advanced Systems Understanding (CASUS), Conrad-Schiedt-Straße 20, 02826 Görlitz, Germany; § Helmholtz Zentrum Dresden-Rossendorf, Bautzner Landstraße 400, 01328 Dresden, Germany; ∥ Institute of Artificial Intelligence, Technische Universität Dresden, Helmholtzstraße 10, 01069 Dresden, Germany

## Abstract

Through-hydrogen-bond scalar couplings are attractive NMR observables because they connect high-precision spectroscopy with local hydrogen-bond structure. It is less clear whether they can also report hydrogen-bond covalency in amorphous ice and other frozen or heterogeneous aqueous environments. Here, we combine ab initio molecular dynamics configurations of water, density functional response calculations of indirect nuclear spin–spin couplings, and absolutely localized molecular orbital (ALMO) energy decomposition analysis. Benchmark calculations against SOPPA­(CCSD) water-dimer references validate BLYP/pcJ-1 for the through-hydrogen-bond ^1h^ *J*_O–H_ coupling. The coupling is dominated by the Fermi contact term and therefore follows an approximately exponential distance dependence, but ensemble and vibrational averaging prevent a transferable one-dimensional distance ruler. Extending earlier NMR/ALMO work on liquid water, ^1h^ *J*_O–H_ correlates with ALMO charge-transfer stabilization and charge-transfer amount. Thus, ^1h^ *J*_O–H_ is an experimentally accessible, covalency-sensitive fingerprint of hydrogen bonds, provided that geometry and ensemble effects are included explicitly.

## Graphical abstract

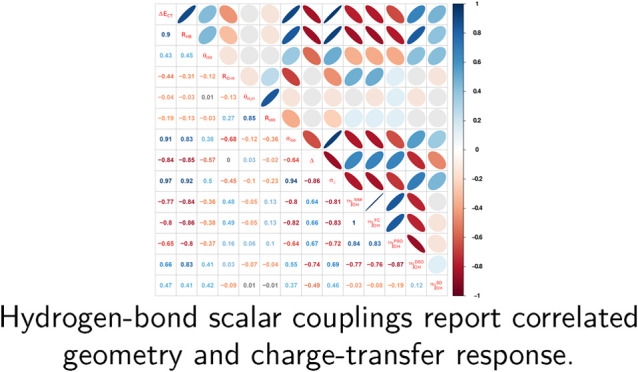

## Introduction

Magnetic-resonance observables provide unusually local information about hydrogen-bonded systems, yet the locality is almost never literal. Hydrogen bonds affect nuclear magnetic shielding tensors, hyperfine tensors, and exchange rates,^1–5^ and an especially direct observable is the indirect nuclear spin–spin coupling transmitted through a hydrogen bond that is stable on the NMR time scale.^6–8^ Such hydrogen-bond scalar couplings (HBCs), commonly written as ^*n*h^ *J*_AB_ with *n* counting all covalent and hydrogen-bond links between nuclei A and B, have been measured in small molecules and biomacromolecules.^9–13^ They can reveal residence times in complexes,^14^ hydrogen-exchange kinetics,^15^ and complete hydrogen-bond networks in favorable cases.^16^ Their broader chemical promise is that a single scalar quantity might encode both a geometric contact and the electronic communication across it. This type of NMR/electronic-structure connection has already been established for liquid water by Elgabarty, Khaliullin, and Kühne, who showed that proton shielding anisotropy correlates with ALMO charge-transfer descriptors and can report hydrogen-bond covalency and its sensitivity to temperature.^3^ Here, we ask whether a different NMR observable, the through-hydrogen-bond scalar coupling, carries an analogous ALMO-defined electronic signal in amorphous-ice-relevant ensembles.

This promise is also the main source of ambiguity. HBCs often decrease approximately exponentially with donor–acceptor or acceptor–hydrogen distance,^17–19^ consistent with the decay of orbital overlap.^20^ However, the experimentally observed coupling is an ensemble average over a nonlinear response property. Hydrogen-bond length, angle, donor O–H stretch, local electric field, and vibrational motion fluctuate together in amorphous and frozen aqueous environments. Consequently, the average scalar coupling is not equivalent to a scalar coupling evaluated at an average hydrogen-bond geometry. The same problem appears in other NMR structure determinations, where vibrational corrections can be significant.^21–23^

The connection to covalency is more subtle still. Hydrogen-bond covalency is not uniquely defined,^24^ and descriptors based on density accumulation, delocalization energy, charge transfer, or kinetic-energy lowering need not be interchangeable.^25–29^ Through-space scalar couplings can also occur without a chemically assigned hydrogen bond,^8,30–33^ and the Fermi contact (FC) contribution reflects spin polarization and exchange correlation rather than literal charge flow.^34,35^ Thus, an HBC should not be treated as a standalone “covalency meter.” The useful question is narrower and experimentally more relevant: after geometric and ensemble effects have been accounted for, does ^1h^ *J*_O–H_ provide a covalency-sensitive fingerprint of hydrogen bonds in amorphous ice?

## Computational Methods

We address this question using disordered water configurations sampled from ab initio molecular dynamics as a broad hydrogen-bond ensemble relevant to amorphous ice and frozen aqueous matrices, together with cluster response calculations of scalar couplings. The ensemble was sampled from a 100 ps ab initio molecular dynamics trajectory of 128 water molecules at ambient density. The same hydrogen bonds are analyzed with absolutely localized molecular orbital (ALMO) energy decomposition and charge-transfer analysis. The ALMO descriptors are periodic CP2K quantities, whereas scalar couplings are evaluated on molecular clusters. For this purpose, we use the periodic condensed-phase extension of the ALMO analysis developed by Kühne and Khaliullin for water and ice,^36,37^ together with its later NMR connection established by Elgabarty, Khaliullin, and Kühne.^3^ This design separates three related but distinct statements: whether ^1h^ *J*_O–H_ is primarily controlled by distance, whether it tracks electronic delocalization across the hydrogen bond, and whether either relation survives the structural disorder relevant to amorphous ice. Computational and theoretical details, including the spin–spin response decomposition and ALMO equations, are given in the Supporting Information.

## Results and Discussion

The first requirement for a useful amorphous-ice analysis is a practical level of theory for many scalar-coupling calculations. Water-dimer scans show that BLYP/pcJ-1 reproduces the SOPPA­(CCSD) reference trend^38–40^ for ^1h^ *J*_O–H_ over the hydrogen-bond distance range relevant to liquid configurations, whereas the same density functional level is less uniformly accurate for all intramolecular couplings (Figures S1–S3). The decomposition of the water-dimer coupling shows that the FC term dominates ^1h^ *J*_O–H_ over the full scan. This explains why ^1h^ *J*_O–H_ has a simple exponential-like distance dependence in idealized geometries. It also limits the interpretation: the dominant response term is strongly distance sensitive, but it is not a pure geometric observable (Figure 1).

**Figure 1:**
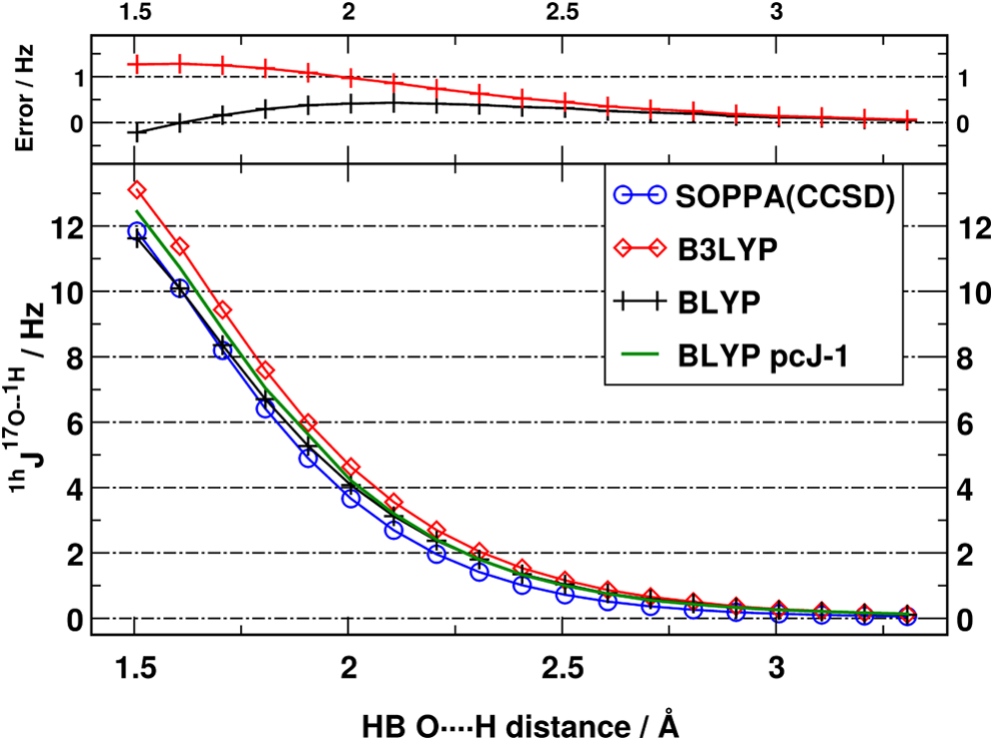
Benchmark of BLYP and B3LYP for calculating ^1h^ *J*_O–H_ as a function of the hydrogen-bond O···H distance. SOPPA­(CCSD) values are used as reference, with errors relative to this reference shown in the upper panel. The green trace corresponds to the production BLYP/pcJ-1 level.

The difference between an idealized distance scan and an amorphous-ice-relevant disordered ensemble is shown in Figure 2. The ALMO charge-transfer stabilization decreases nearly exponentially with the hydrogen-bond O···H distance, as expected for an orbital-delocalization descriptor. The scalar coupling follows the same qualitative trend but with larger scatter and systematic deviations. Representative averages illustrate the size of the effect (Table 1): ^1h^ *J*_O–H_ increases from about 4.8 Hz in the optimized dimer to 5.3 Hz after vibrational averaging and to 5.9 Hz in the disordered trajectory ensemble. Here, the optimized dimer is the B3LYP/def2-TZVP minimum used for the SOPPA­(CCSD) reference calculations, the vibrationally averaged dimer samples nuclear fluctuations around this reference, and the trajectory ensemble consists of first-solvation-shell clusters extracted from the periodic 100 ps ab initio molecular dynamics trajectory. By contrast, the intramolecular ^1^ *J*_O–H_ and ^1^ *J*_H–H_ couplings change only weakly with hydrogen-bond donation. Thus, ^1h^ *J*_O–H_ is the most direct scalar-coupling reporter of the intermolecular contact, but it is also the most exposed to the fluctuations that make one-dimensional distance calibration unreliable.

**Figure 2:**
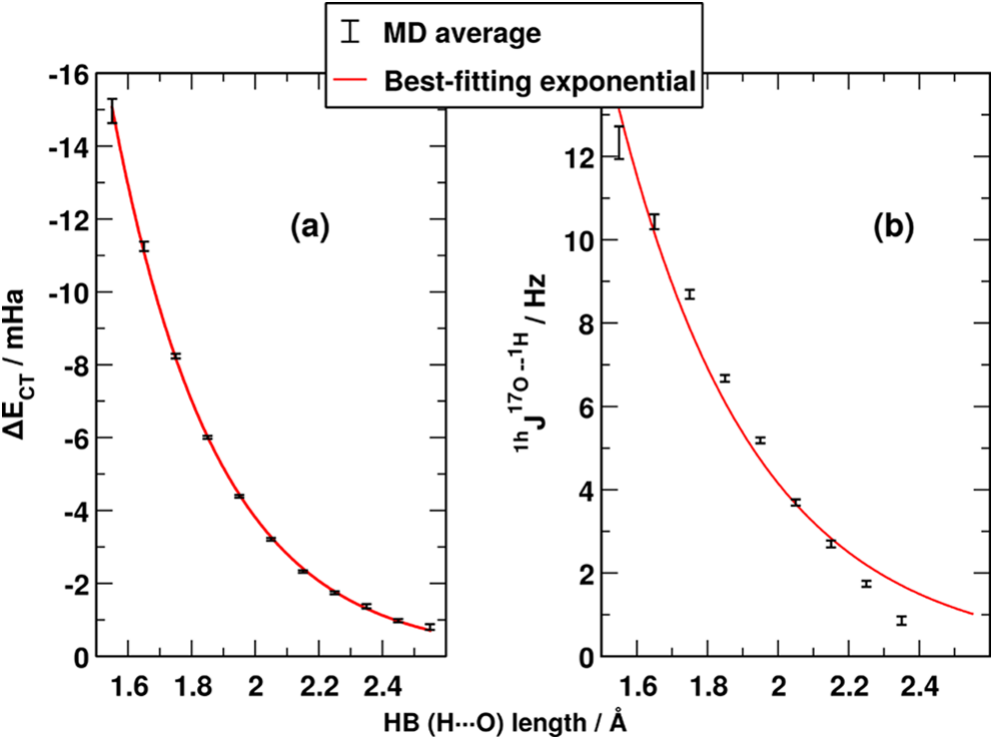
Binned ensemble averages of (a) ALMO charge-transfer stabilization and (b) ^1h^ *J*_O–H_ as functions of hydrogen-bond length. Both observables decay with increasing O···H distance, but the scalar coupling displays larger scatter and more structured deviations from a simple exponential model.

**Table 1:** Representative BLYP/pcJ-1 Scalar Couplings for the Water Dimer and the Condensed-Phase Trajectory Ensemble

geometry	^1h^ *J*_O–H_ (Hz)	^1^ *J*_O–H_ (Hz)	^1^ *J*_H–H_ (Hz)
optimized dimer	4.8	–71.4	–6.0
vibrationally averaged dimer	5.3	–69.0	–6.0
trajectory mean	5.9	–70.0	–6.0

Figure 3 tests the covalency connection more directly. The FC contribution to ^1h^ *J*_O–H_ correlates with both ALMO charge-transfer amount and charge-transfer stabilization, with correlation coefficients of about 0.72–0.82 depending on the descriptor. This level of correlation is chemically meaningful because the two quantities are different response properties: ALMO charge transfer measures the energetic and density response obtained by relaxing a block-localized electronic structure, whereas the FC term probes spin polarization induced by nuclear magnetic perturbations. The fact that they correlate across a disordered ensemble indicates that both are governed by electronic communication across the hydrogen bond. The point-to-point dispersion is equally important. It shows that ^1h^ *J*_O–H_ is covalency-sensitive, not covalency identical, because hydrogen-bond length, hydrogen-bond angle, donor O–H stretch, and electronic polarization fluctuate together in the condensed-phase ensemble.

**Figure 3:**
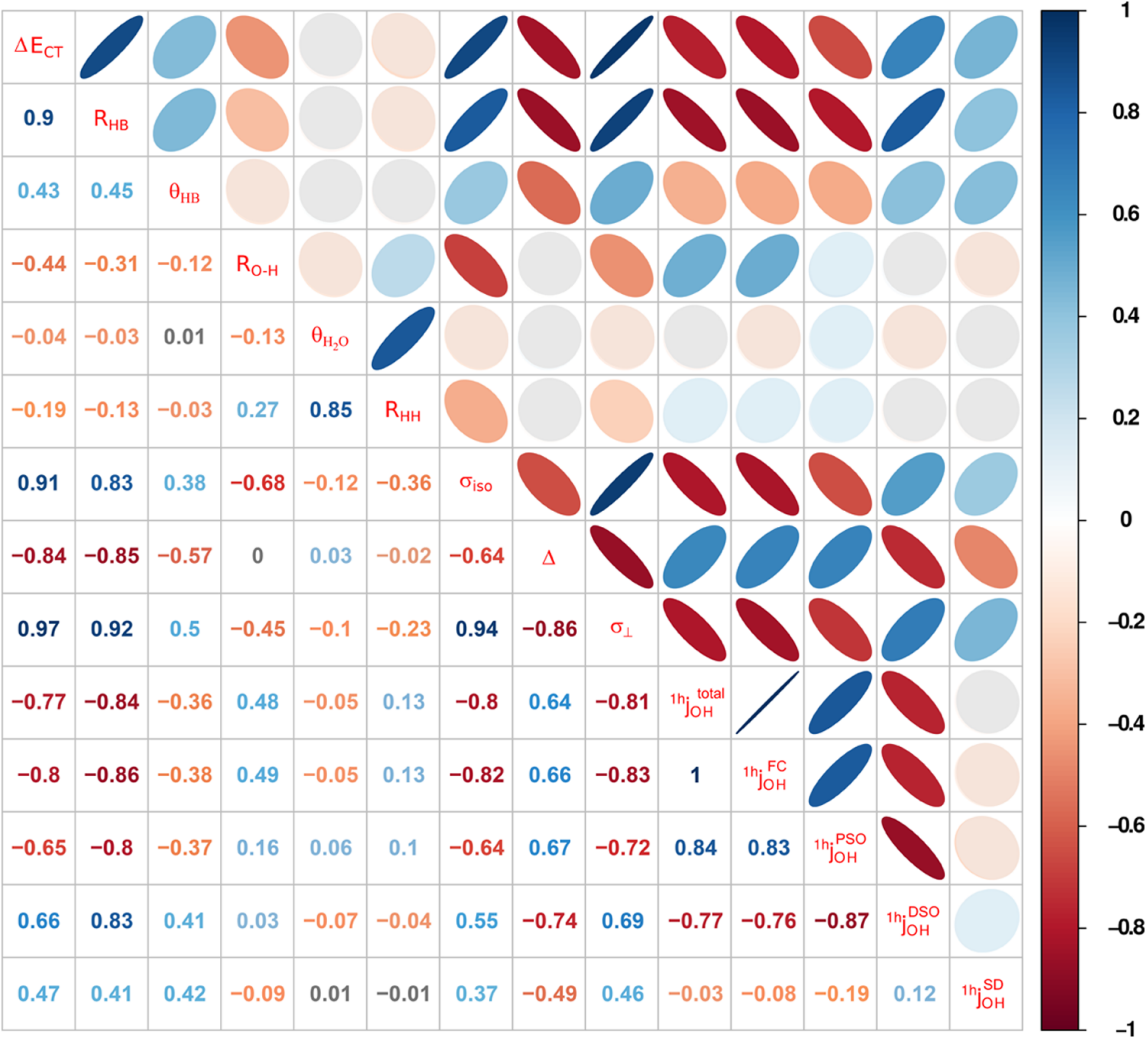
Correlation matrix for the FC contribution to ^1h^ *J*_O–H_ and the ALMO, geometry, shielding, and coupling descriptors. The lower triangle gives Pearson correlation coefficients, and the upper triangle represents the same correlations as ellipses. Descriptor labels follow the notation defined in the Supporting Information.

The residuals in Figure 4 explain why this distinction matters. If ^1h^ *J*_O–H_ were only a noisy function of hydrogen-bond length, the residuals of a distance model would be random. Instead, they retain structure, reflecting coupled fluctuations of hydrogen-bond angle, donor O–H stretch, and local environment. Log–linear regression worsens this problem by unevenly weighting short and long hydrogen bonds, and adding low-order polynomial corrections gives only marginal improvement. The limitation is therefore physical rather than purely statistical. For amorphous ice, a scalar coupling measured or computed for an ensemble should be interpreted as a calibrated fingerprint of persistent close contacts and correlated electronic response, not as a direct distance ruler.

**Figure 4:**
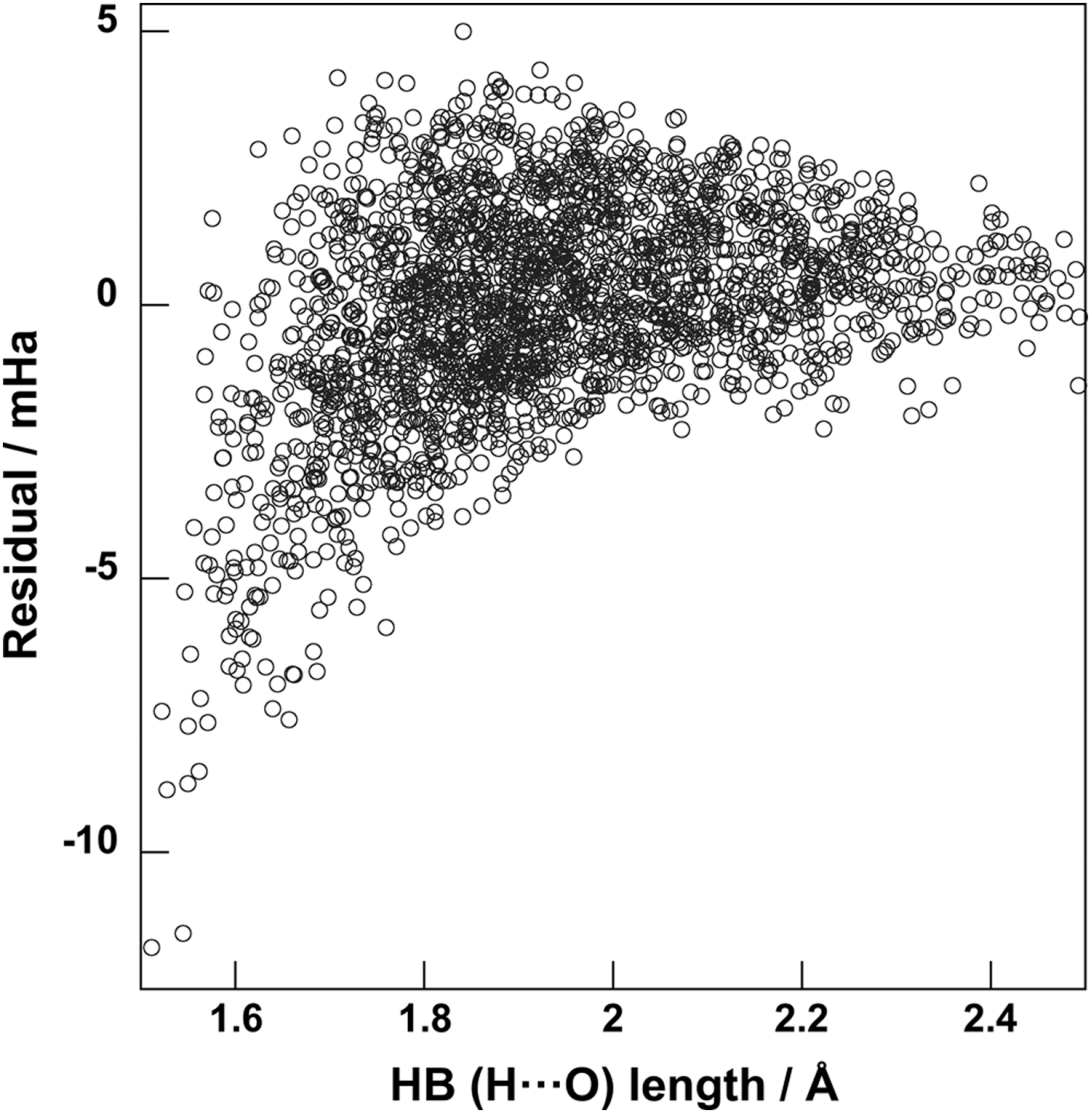
Residuals of a linear fit between ALMO charge-transfer energy and ^1h^ *J*_O–H_ plotted as a function of hydrogen-bond length.

The central point is that ^1h^ *J*_O–H_ probes hydrogen-bond electronic communication in a structurally disordered, amorphous-ice-relevant ensemble. ^1h^ *J*_O–H_ is a two-center magnetic-response property transmitted through the donor–hydrogen–acceptor pathway; its correlation with ALMO charge transfer therefore links an experimentally accessible coupling to the electronic component of a specific intermolecular contact. More generally, the analysis suggests a practical NMR strategy for disordered hydrogen-bonded systems: use response properties not as single-coordinate structural rulers, but as ensemble-calibrated observables linked to electronic-structure descriptors. This makes the observable useful as an ensemble-calibrated fingerprint of persistent hydrogen bonds in amorphous ice and frozen aqueous matrices, where individual liquid-like hydrogen bonds are not directly resolvable.

## Conclusions

In summary, through-hydrogen-bond scalar couplings in water are neither purely geometric observables nor direct measurements of charge transfer. They are, however, experimentally accessible and covalency-sensitive. The FC term controls the distance trend, structural fluctuations broaden it, and ALMO analysis shows that the remaining electronic signal correlates with charge-transfer stabilization across the hydrogen bond. The resulting picture is useful precisely because it is calibrated rather than literal: ^1h^ *J*_O–H_ can be used to infer hydrogen-bond charge-transfer character semiquantitatively, provided that geometric and ensemble effects are modeled explicitly.

## Supplementary Material



## Notes

Computational details, spin–spin response and ALMO charge-transfer equations, water-dimer benchmark calculations, coupling-component decompositions, and basis-set convergence tests (PDF)
