# Supplementary material for: Hydrogen-Bond Scalar Couplings as Covalency-Sensitive NMR Fingerprints of Amorphous Ice
Source: J Phys Chem B. 2026 Jul 24;130(31):8061–5. doi: 10.1021/acs.jpcb.6c03372 (PMC13455050; doi:10.1021/acs.jpcb.6c03372)
Supplement: Supplementary file 1 [file jp6c03372_si_001.pdf]

Supporting Information: Supporting Information for: Hydrogen-Bond Scalar Couplings as Covalency-Sensitive NMR Fingerprints of Amorphous Ice

Hossam Elgabarty[*,†] and Thomas D. Kühne[‡]

†*Department of Chemistry, University of Paderborn, Warburger Str. 100, D-33098 Paderborn, Germany*

‡*Center for Advanced Systems Understanding (CASUS), Conrad-Schiedt-Straße 20, 02826 Görlitz*

¶*Helmholtz Zentrum Dresden-Rossendorf, Bautzner Landstraße 400, 01328 Dresden, Germany*

§*Institute of Artificial Intelligence, Technische Universität Dresden, Helmholtzstraße 10, 01069 Dresden, Germany*

E-mail: hossam.elgabarty@uni-paderborn.de

This Supporting Information provides the theoretical and computational details underlying the Article. Subsections S1.1–S1.4 define the molecular-dynamics sampling, NMR response calculations, spin–spin coupling decomposition, and absolutely localized molecular orbital (ALMO) charge-transfer descriptors used to interpret the main-text correlations. Figures S1–S4 document the water-dimer reference calculations, coupling-component decomposition, and basis-set tests that justify the production BLYP/pcJ-1 scalar-coupling calculations. The descriptors used in the main-text correlation matrix are defined as follows: ΔE_{CT} is the ALMO charge-transfer stabilization, R_{HB} is the hydrogen-bond length, θ_{HB} is the hydrogen-bond angle, $R_{\mathrm{O-H}}$ is the covalent O–H bond length, $\theta_{\mathrm{H_2O}}$ is the intramolecular H–O–H angle, and R_{HOH} is the intramolecular H–H distance. The shielding descriptors σ_{iso}, Δ, and $\sigma_{\perp}$ denote the isotropic shielding, shielding anisotropy, and the average of the two principal components in the plane orthogonal to the hydrogen bond, respectively. The scalar-coupling labels denote the total ${}^{1h}J_{O-H}$ coupling and its response contributions defined in Section S1.3.

S1 Computational and Theoretical Details

S1.1 Molecular dynamics and cluster construction

Ab initio molecular dynamics simulations were performed for a periodic cubic cell containing 128 water molecules at ambient density using the second-generation Car–Parrinello molecular-dynamics method of Kühne and coworkers.[S1–S3] The production trajectory was restarted from a pre-equilibrated constant-volume trajectory at $T = 298\,\mathrm{K}$ and extended for 100 ps. Energies and forces were computed with the mixed Gaussian and plane-wave approach,[S4] using the BLYP exchange-correlation functional, Grimme's D3 dispersion correction,[S5] a TZV2P orbital basis,[S6] and a density cutoff of 600 Ry. All molecular dynamics calculations were carried out with the QUICKSTEP module of CP2K.[S7,S8] Hydrogen bonds were identified by the geometric criterion $R_{\mathrm{OO}} < 3.5\,\text{Å}$ and $\angle\mathrm{OHO} < 30^\circ$.[S9]

Ten configurations were extracted at regular intervals from the trajectory. For each water molecule in each configuration, a molecular cluster was built from the central molecule and all water molecules whose oxygen atoms were within 3.5 Å of the central oxygen. Scalar couplings were evaluated only between nuclei in the central molecule and its neighbors. This reduces the number of response calculations while retaining the first-shell couplings used in the hydrogen-bond analysis.

S1.2 NMR response calculations

Nuclear magnetic shieldings were computed in CP2K under periodic boundary conditions using the all-electron Gaussian and augmented-plane-wave (GAPW) method[S10,S11] with a 600 Ry density cutoff and the aug-pcSseg-2 basis set.[S12] These shielding calculations are fully periodic: the perturbed current density, magnetic susceptibility, and final shielding tensors are evaluated in the simulation cell. This is distinct from the scalar-coupling calculations described below, where molecular clusters are used in DALTON. The CP2K implementation follows a magnetic linear-response formulation in which the first-order current density $\mathbf{j}_\beta^{(1)}(\mathbf{r})$ induced by a homogeneous magnetic field along direction β is evaluated on the periodic grid. In GAPW form, the response current can be viewed as a smooth GPW current plus atom-centered all-electron reconstruction terms,

$$\mathbf{j}_\beta^{(1)}(\mathbf{r}) = \tilde{\mathbf{j}}_\beta^{(1)}(\mathbf{r}) + \sum_A \left[\mathbf{j}_{A,\beta}^{(1),\mathrm{AE}}(\mathbf{r}) - \tilde{\mathbf{j}}_{A,\beta}^{(1)}(\mathbf{r})\right], \tag{1}$$

where the tilde denotes the smooth pseudo-current and A runs over atom-centered augmentation regions. For a nucleus at $\mathbf{R}_K$, the microscopic shielding contribution can be written in atomic units as

$$\sigma_{\alpha\beta}^{\mathrm{mic}}(\mathbf{R}_K) = -\frac{\alpha_{\mathrm{fs}}^2}{2} \int_\Omega \frac{\left[(\mathbf{r}-\mathbf{R}_K) \times \mathbf{j}_\beta^{(1)}(\mathbf{r})\right]_\alpha}{|\mathbf{r}-\mathbf{R}_K|^3}\, d\mathbf{r}, \tag{2}$$

where $\alpha_{\rm fs}$ is the fine-structure constant and Ω is the simulation cell. The GAPW augmentation generates the corresponding local correction

$$\Delta\sigma^{\rm GAPW}_{\alpha\beta}(\mathbf{R}_K) = -\frac{\alpha_{\rm fs}^2}{2}\sum_A \int_{\Omega_A} \frac{\left[(\mathbf{r}-\mathbf{R}_K)\times\left(\mathbf{j}^{(1),\rm AE}_{A,\beta}(\mathbf{r})-\tilde{\mathbf{j}}^{(1)}_{A,\beta}(\mathbf{r})\right)\right]_\alpha}{|\mathbf{r}-\mathbf{R}_K|^3}\, d\mathbf{r}, \tag{3}$$

which is numerically integrated on atom-centered grids close to the nuclei. In periodic boundary conditions the shielding reported for an atom is the microscopic contribution plus a macroscopic susceptibility term,

$$\sigma_{\alpha\beta}(\mathbf{R}_K) = \sigma^{\rm mic}_{\alpha\beta}(\mathbf{R}_K) + \Delta\sigma^{\rm GAPW}_{\alpha\beta}(\mathbf{R}_K) + \sigma^{\rm mac}_{\alpha\beta}(\boldsymbol{\chi}), \tag{4}$$

where $\boldsymbol{\chi}$ is the periodic-cell magnetic susceptibility tensor and the last term accounts for the long-wavelength magnetic boundary condition.[S11] Thus the CP2K shielding calculation retains both the extended condensed-phase current response and the local all-electron near-nuclear contribution that is essential for NMR shieldings. Indirect spin–spin couplings were computed with DALTON[S13] using BLYP and B3LYP exchange-correlation functionals together with Jensen's pcJ basis sets.[S14] The production scalar-coupling calculations used BLYP/pcJ-1, benchmarked against SOPPA(CCSD) reference calculations.[S15–S17]

S1.3 Spin–spin coupling decomposition

In the nonrelativistic limit, the reduced spin–spin coupling tensor is the sum of diamagnetic spin-orbit (DSO), paramagnetic spin-orbit (PSO), Fermi-contact (FC), and spin-dipolar (SD) response contributions. The leading relativistic corrections are negligible for water.[S18] The one-electron perturbation operators for nucleus K may be written as

$$\hat{h}^{\rm FC}_K = \frac{8\pi\alpha^2}{3}\sum_i \delta(\mathbf{r}_{iK})\mathbf{s}_i, \tag{5}$$

$$\hat{h}_K^{\text{SD}} = \alpha^2 \sum_i \frac{3(\mathbf{r}_{iK} \cdot \mathbf{s}_i)\mathbf{r}_{iK} - r_{iK}^2 \mathbf{s}_i}{r_{iK}^5}, \tag{6}$$

$$\hat{h}_K^{\text{PSO}} = -i\alpha^2 \sum_i \frac{\mathbf{r}_{iK} \times \nabla_i}{r_{iK}^3}, \tag{7}$$

and

$$\hat{h}_{KL}^{\text{DSO}} = \alpha^4 \sum_i \frac{(\mathbf{r}_{iK} \cdot \mathbf{r}_{iL})\mathbf{1} - \mathbf{r}_{iK}\mathbf{r}_{iL}^T}{r_{iK}^3 r_{iL}^3}. \tag{8}$$

Within second-order response theory, the reduced, gyromagnetic-ratio-independent coupling tensor is[S19]

$$\begin{aligned} \mathbf{K}_{KL} =& \langle 0|\hat{h}_{KL}^{\text{DSO}}|0\rangle + \langle\langle \hat{h}_K^{\text{PSO}}; (\hat{h}_L^{\text{PSO}})^T \rangle\rangle_{\omega=0} \\ &+ \langle\langle \hat{h}_K^{\text{FC}} + \hat{h}_K^{\text{SD}}; (\hat{h}_L^{\text{FC}} + \hat{h}_L^{\text{SD}})^T \rangle\rangle_{\omega=0}. \end{aligned} \tag{9}$$

The DSO term is a ground-state expectation value, whereas the PSO term is obtained from singlet response equations and the FC and SD terms from triplet response equations. The scalar coupling in frequency units is obtained from the isotropic component of $\mathbf{K}_{KL}$,

$$J_{KL} = \frac{h\gamma_K\gamma_L}{4\pi^2} \frac{\text{Tr}(\mathbf{K}_{KL})}{3}. \tag{10}$$

Throughout this work, the gyromagnetic ratios of ^{1}H and ^{17}O are used.

S1.4 ALMO charge-transfer descriptors

The charge-transfer descriptors were obtained with the absolutely localized molecular orbital (ALMO) energy-decomposition and charge-transfer machinery. The original molecular ALMO framework[S20,S21] was extended by Kühne and Khaliullin for periodic condensed-phase water and ice,[S22,S23] and subsequently used by Elgabarty, Khaliullin, and Kühne to connect ALMO charge-transfer descriptors with NMR observables in liquid water.[S24] In this formulation the occupied orbitals are first constrained to remain block-localized on molecular fragments. The ALMO descriptors used here are therefore periodic CP2K quantities,

not cluster post-processing results. Each water molecule defines one fragment/domain in the periodic supercell, while the overlap, density, and Kohn–Sham matrices retain the electrostatic and Pauli environment of the condensed phase through the ordinary QUICKSTEP self-consistent field. In the Γ-point supercell notation used here, block localization imposes

$$(T_0)_{\mu i} = 0 \qquad \text{if} \quad \mu \notin D(i), \tag{11}$$

where $D(i)$ is the atomic-orbital domain of occupied orbital i. The subsequent extended-ALMO step relaxes this constraint only for the donor–acceptor blocks allowed by the CP2K delocalization mask. If T_0 denotes the coefficient matrix of the block-diagonal ALMOs and S is the AO overlap matrix, the ALMO overlap and density matrix are

$$\sigma_0 = T_0^T S T_0, \qquad P_0 = f\, T_0 \sigma_0^{-1} T_0^T, \tag{12}$$

where f is the spin occupation factor, $f = 2$ for the closed-shell calculations considered here. Delocalization is then introduced by allowing an extended-ALMO coefficient matrix $T = T_0 + X$ and the corresponding density

$$\sigma = T^T S T, \qquad P = f\, T \sigma^{-1} T^T. \tag{13}$$

The CP2K analysis evaluates the charge-transfer stabilization from the single-excitation amplitudes X and the virtual–occupied projected Kohn–Sham matrix of the block-localized reference,

$$F_{vo}^{(0)} = (1 - S R_0)\, F^{(0)} T_0 \sigma_0^{-1}, \qquad R_0 = T_0 \sigma_0^{-1} T_0^T, \tag{14}$$

as

$$\Delta E_{\text{CT}} = \sum_s f_s \operatorname{Tr}\left[X_s^T F_{vo,s}^{(0)} \right]. \tag{15}$$

The pair-resolved ALMO energy terms are fragment-block sums of the element-wise product $X_s \circ F^{(0)}_{vo,s}$,

$$\Delta E_{\mathrm{CT}}(A \leftarrow B) = \sum_s f_s \sum_{\mu \in A} \sum_{i \in B} X_{\mu i,s} \left(F^{(0)}_{vo,s}\right)_{\mu i} . \tag{16}$$

The associated charge-transfer analysis uses the projected density response

$$Q_s = (1 - SR_{0,s})\, S X_s \sigma_s^{-1}, \tag{17}$$

and forms fragment-block sums of $X_s \circ Q_s$,

$$q_{\mathrm{CT}}(A \leftarrow B) = \sum_s f_s \sum_{\mu \in A} \sum_{i \in B} X_{\mu i,s} \left(Q_s\right)_{\mu i} . \tag{18}$$

Periodic images enter these descriptors through the self-consistent periodic matrices S, $F^{(0)}$, and P; the reported pair terms are the molecular fragment-block contributions in the primary simulation cell. Thus the ALMO energy and charge descriptors used here are computed from the same relaxation of the block-localized reference, but they measure an energy lowering and an amount of transferred density, respectively.

S2 Benchmark and Supporting Figures

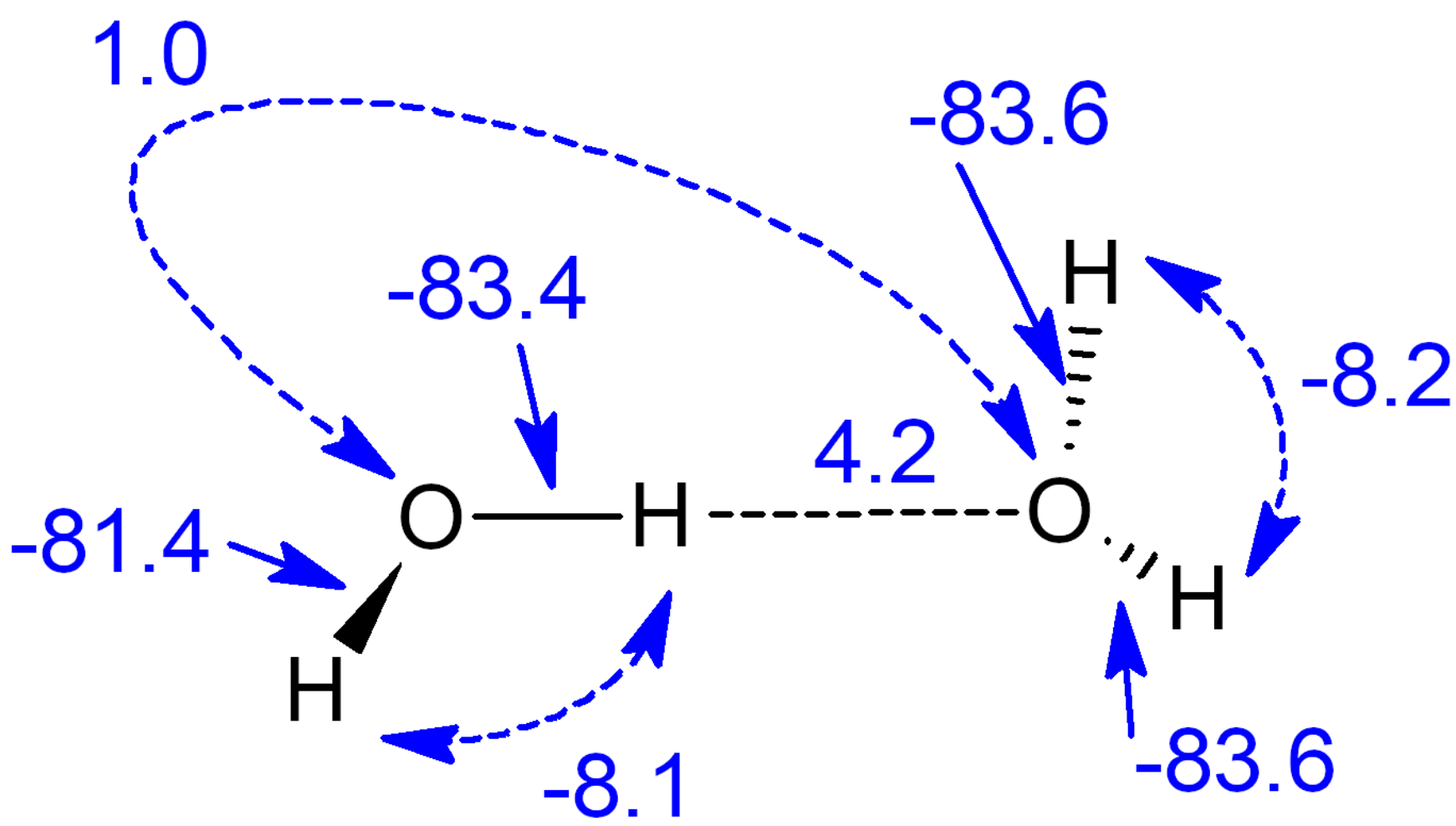

Figure S1: j-couplings in the optimized water dimer calculated with SOPPA(CCSD)/pcJ-2. All values are in Hertz. Geometry was optimized with B3LYP and Ahlrichs' def2-TZVP basis set.

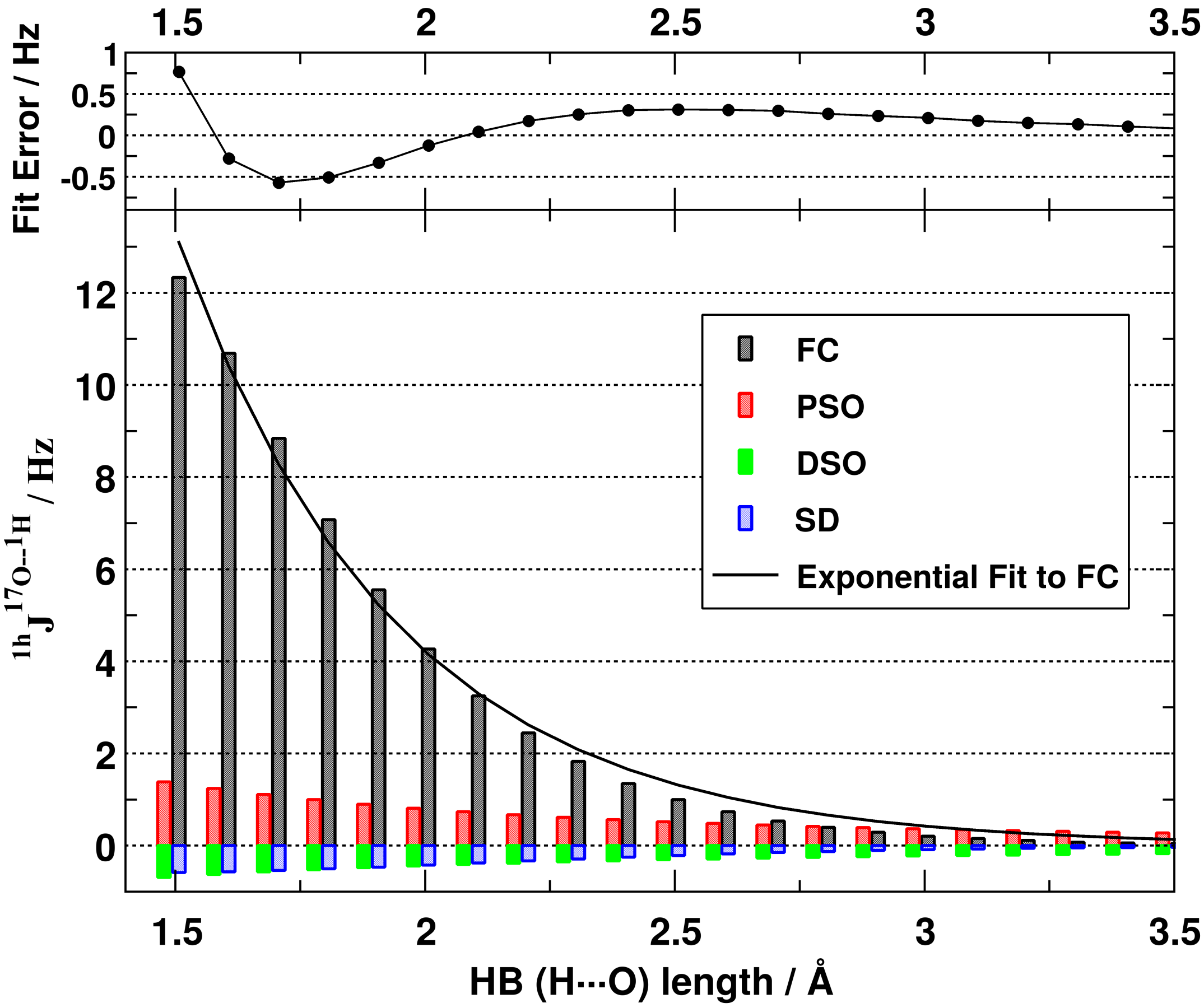

Figure S2: Dependence of the four response contributions to ${}^{1h}J_{O-H}$ on the hydrogen-bond O$\cdots$H distance at the BLYP/pcJ-1 level. The geometries were generated from the optimized water dimer by varying the hydrogen-bond length while keeping the monomer geometries rigid.

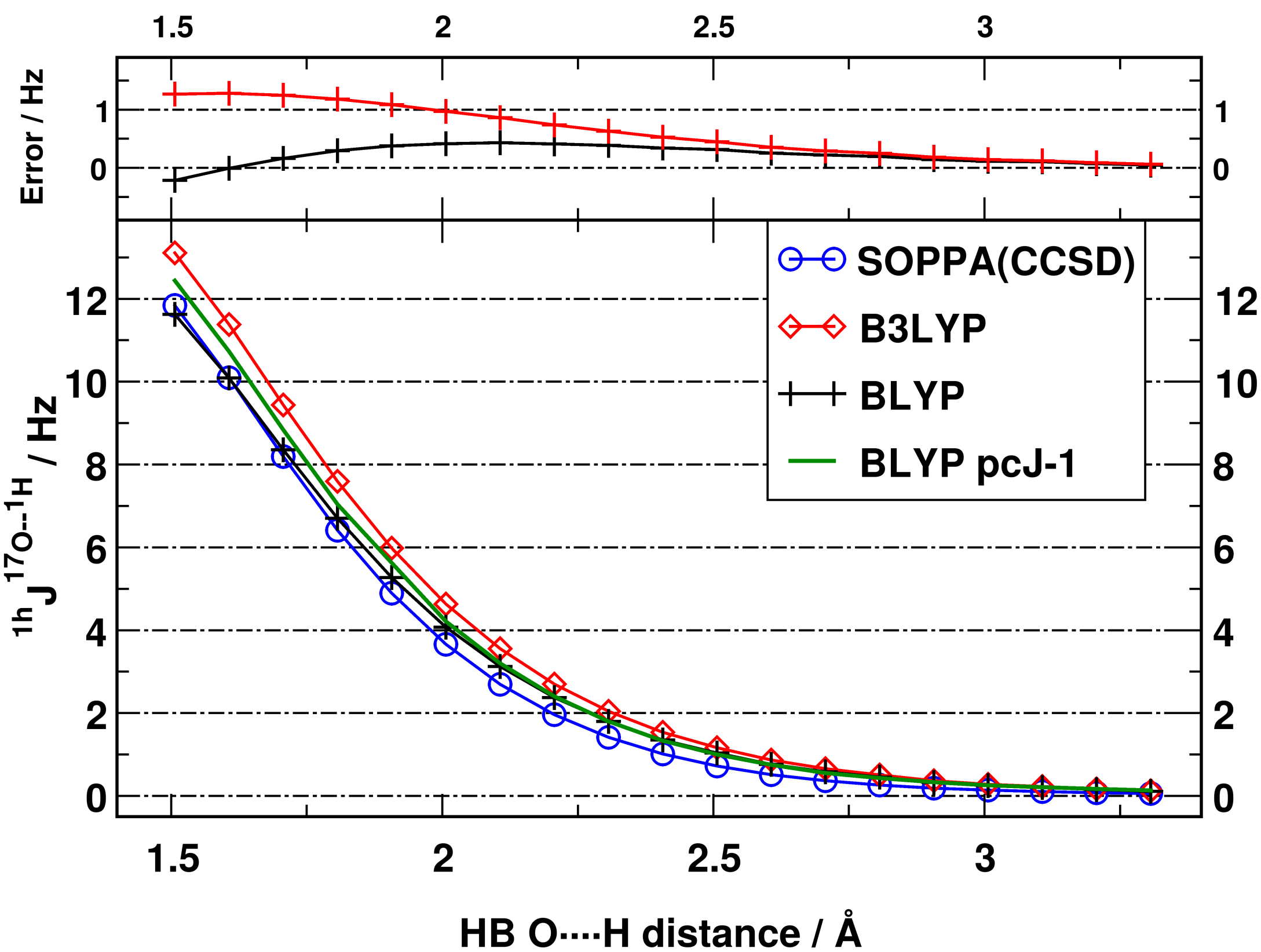

Figure S3: Accuracy of $^{1h}J_{O-H}$ obtained from BLYP (black) and B3LYP (red) as compared to SOPP(CCSD) (blue), all using the pcJ-2 basis set. The green line shows that the performance of BLYP/pcJ-1 is only very slightly inferior to BLYP/pcJ-2.

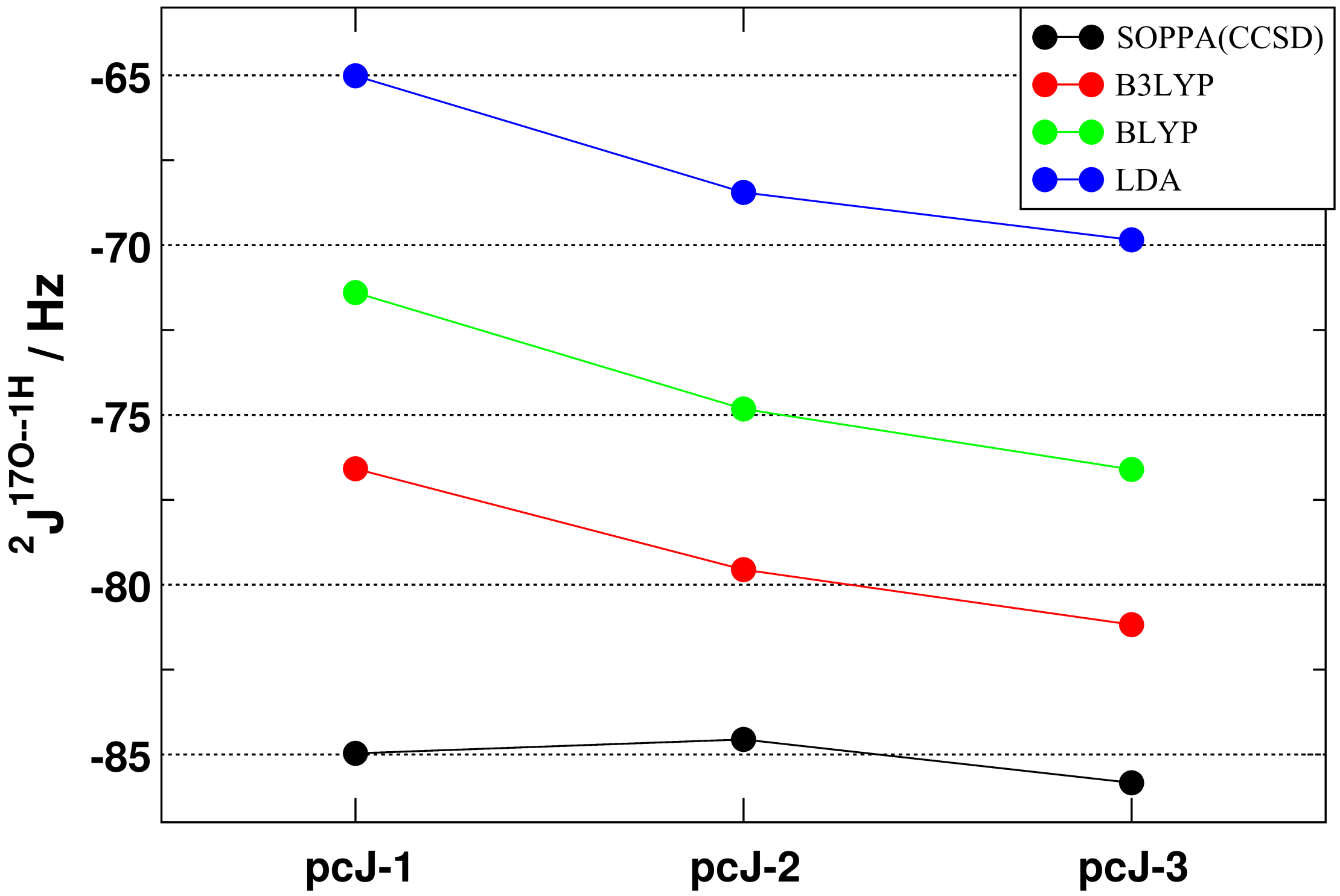

Figure S4: Basis set convergence of $^{1}J_{O-H}$, $^{1}J_{H-H}$, and $^{1h}J_{O-H}$. Taking SOPPA(CCSD) values as our benchmark, BLYP outperforms B3LYP in the accuracy of $^{1h}J_{O-H}$, this is unlike the situation with intramolecular j-couplings which follow the previously reported accuracy trend, in parallel to the "functional ladder": LDA $\rightarrow$ GGA $\rightarrow$ Hybrid. Panel 1: $^{1}J_{O-H}$.

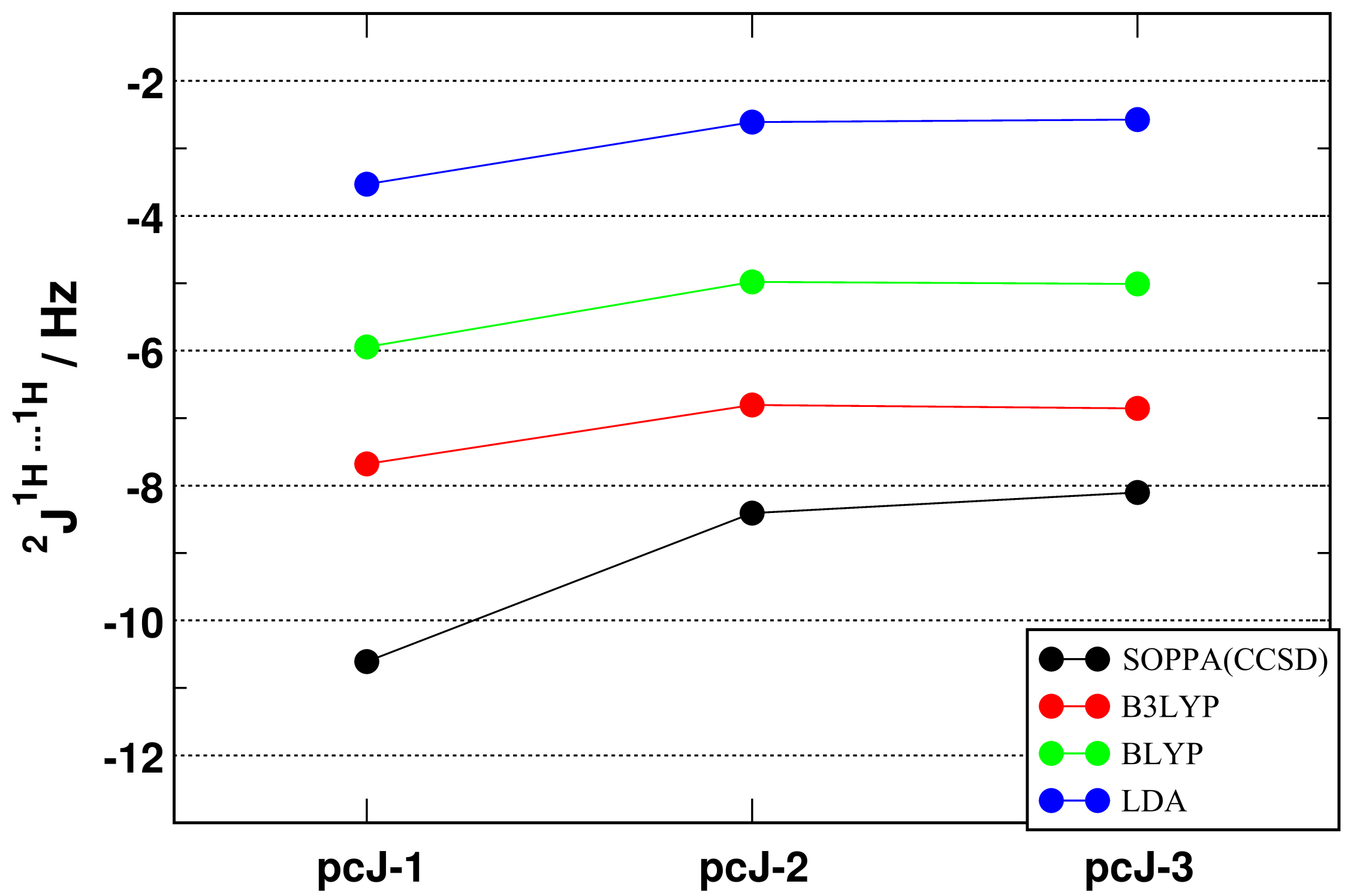

Figure S4: Figure S4 continued. Panel 2: ${}^{1}J_{H-H}$.

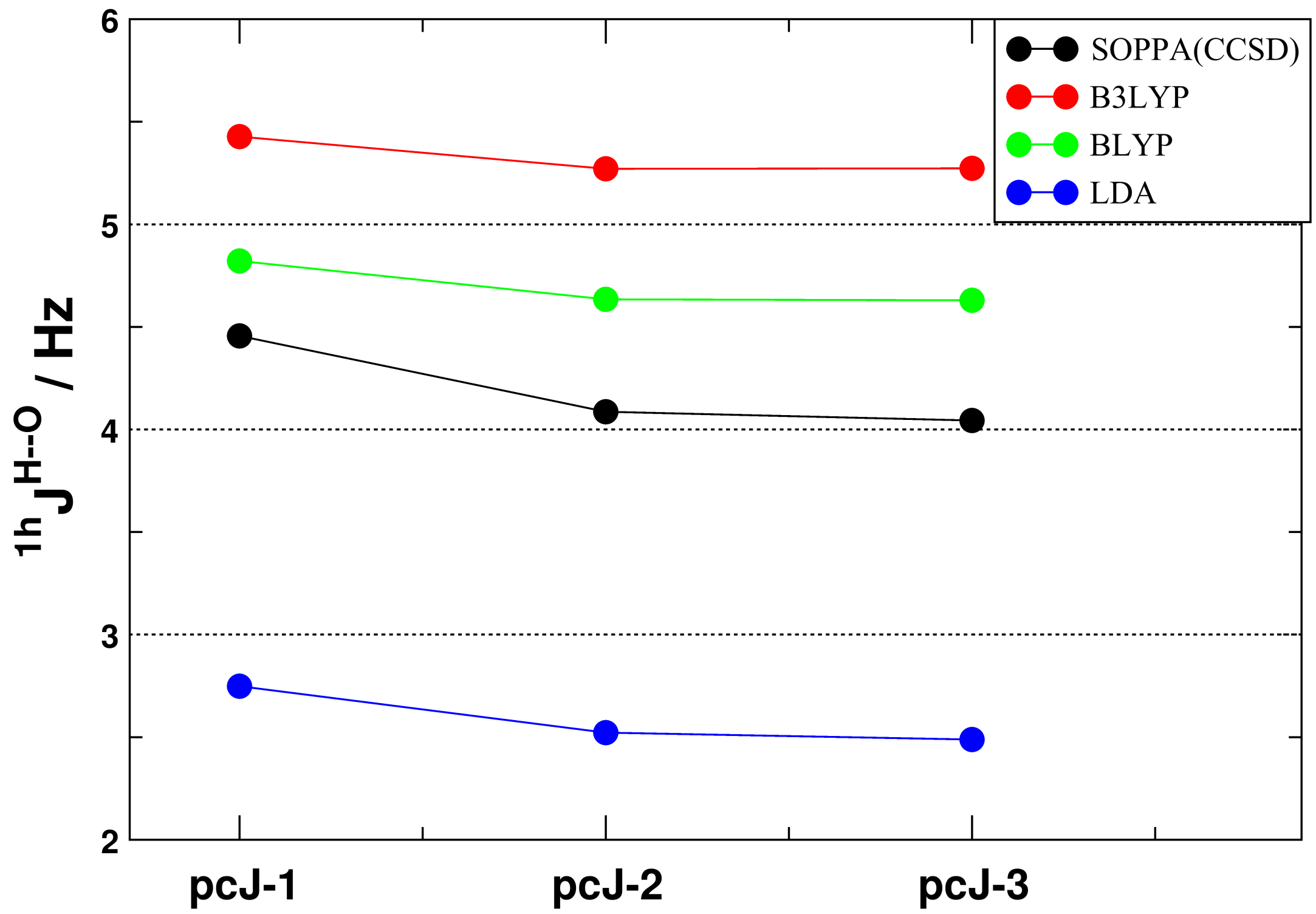

Figure S4: Figure S4 continued. Panel 3: $^{1h}J_{O-H}$.

References

(S1) Kühne, T. D.; Krack, M.; Mohamed, F. R.; Parrinello, M. Efficient and Accurate Car-Parrinello-like Approach to Born-Oppenheimer Molecular Dynamics. *Phys. Rev. Lett.* **2007**, *98*, 066401.

(S2) Kühne, T. D. Second Generation Car-Parrinello Molecular Dynamics. *WIREs Comput. Mol. Sci.* **2014**, *4*, 391–406.

(S3) Hutter, J.; Iannuzzi, M.; Kühne, T. D. *Reference Module in Chemistry, Molecular Sciences and Chemical Engineering*; Elsevier, 2023.

(S4) Lippert, G.; Hutter, J.; Parrinello, M. A Hybrid Gaussian and Plane Wave Density Functional Scheme. *Mol. Phys.* **1997**, *92*, 477–488.

(S5) Grimme, S.; Antony, J.; Ehrlich, S.; Krieg, H. A Consistent and Accurate Ab Initio Parametrization of Density Functional Dispersion Correction (DFT-D) for the 94 Elements H-Pu. *J. Chem. Phys.* **2010**, *132*, 154104.

(S6) VandeVondele, J.; Hutter, J. Gaussian Basis Sets for Accurate Calculations on Molecular Systems in Gas and Condensed Phases. *J. Chem. Phys.* **2007**, *127*, 114105.

(S7) Kühne, T. D.; Iannuzzi, M.; Del Ben, M.; Rybkin, V. V.; Seewald, P.; Stein, F.; Laino, T.; Khaliullin, R. Z.; Schütt, O.; Schiffmann, F.; Golze, D.; Wilhelm, J.; Chulkov, S.; Bani-Hashemian, M. H.; Weber, V.; Borštník, U.; Taillefumier, M.; Jakobovits, A. S.; Lazzaro, A.; Pabst, H.; Müller, T.; Schade, R.; Guidon, M.; Andermatt, S.; Holmberg, N.; Schenter, G. K.; Hehn, A.; Bussy, A.; Belleflamme, F.; Tabacchi, G.; Glöß, A.; Lass, M.; Bethune, I.; Mundy, C. J.; Plessl, C.; Watkins, M.; VandeVondele, J.; Krack, M.; Hutter, J. CP2K: An Electronic Structure and Molecular Dynamics Software Package—Quickstep: Efficient and Accurate Electronic Structure Calculations. *J. Chem. Phys.* **2020**, *152*, 194103.

(S8) Iannuzzi, M.; Wilhelm, J.; Stein, F.; Bussy, A.; Elgabarty, H.; Golze, D.; Hehn, A.-S.; Graml, M.; Marek, S.; Sertcan Gökmen, B.; Schran, C.; Forbert, H.; Khaliullin, R. Z.; Kozhevnikov, A.; Taillefumier, M.; Meli, R.; Rybkin, V. V.; Brehm, M.; Schade, R.; Schütt, O.; Pototschnig, J. V.; Mirhosseini, H.; Knüpfer, A.; Marx, D.; Krack, M.; Hutter, J.; Kühne, T. D. The CP2K Program Package Made Simple. *J. Phys. Chem. B* **2026**, *130*, 1237–1310.

(S9) Luzar, A.; Chandler, D. Hydrogen-bond Kinetics in Liquid Water. *Nature* **1996**, *379*, 55–57.

(S10) Lippert, G.; Hutter, J.; Parrinello, M. The Gaussian and Augmented-plane-wave Density Functional Method for Ab Initio Molecular Dynamics Simulations. *Theor. Chem. Acc.* **1999**, *103*, 124–140.

(S11) Weber, V.; Iannuzzi, M.; Giani, S.; Hutter, J.; Declerck, R.; Waroquier, M. Magnetic Linear Response Properties Calculations with the Gaussian and Augmented-plane-wave Method. *J. Chem. Phys.* **2009**, *131*, 014106.

(S12) Jensen, F. Segmented Contracted Basis Sets Optimized for Nuclear Magnetic Shielding. *J. Chem. Theory Comput.* **2015**, *11*, 132–138.

(S13) Aidas, K.; Angeli, C.; Bak, K. L.; Bakken, V.; Bast, R.; Boman, L.; Christiansen, O.; Cimiraglia, R.; Coriani, S.; Dahle, P.; Dalskov, E. K.; Ekstrom, U.; Enevoldsen, T.; Eriksen, J. J.; Ettenhuber, P.; Fernandez, B.; Ferrighi, L.; Fliegl, H.; Frediani, L.; Hald, K.; Halkier, A.; Hattig, C.; Heiberg, H.; Helgaker, T.; Hennum, A. C.; Hettema, H.; Hjertenaes, E.; Host, S.; Hoyvik, I.-M.; Iozzi, M. F.; Jansik, B.; Jensen, H. J. A.; Jonsson, D.; Jorgensen, P.; Kauczor, J.; Kirpekar, S.; Kjaergaard, T.; Klopper, W.; Knecht, S.; Kobayashi, R.; Koch, H.; Kongsted, J.; Krapp, A.; Kristensen, K.; Ligabue, A.; Lutnaes, O. B.; Melo, J. I.; Mikkelsen, K. V.; Myhre, R. H.; Neiss, C.; Nielsen, C. B.; Norman, P.; Olsen, J.; Olsen, J. M. H.; Osted, A.; Packer, M. J.;

Pawlowski, F.; Pedersen, T. B.; Provasi, P. F.; Reine, S.; Rinkevicius, Z.; Ruden, T. A.; Ruud, K.; Rybkin, V. V.; Salek, P.; Samson, C. C. M.; de Meras, A. S.; Saue, T.; Sauer, S. P. A.; Schimmelpfennig, B.; Sneskov, K.; Steindal, A. H.; Sylvester-Hvid, K. O.; Taylor, P. R.; Teale, A. M.; Tellgren, E. I.; Tew, D. P.; Thorvaldsen, A. J.; Thogersen, L.; Vahtras, O.; Watson, M. A.; Wilson, D. J. D.; Ziolkowski, M.; Agren, H. The Dalton quantum chemistry program system. *WIREs Comput. Mol. Sci.* **2014**, *4*, 269–284.

(S14) Jensen, F. The Optimum Contraction of Basis Sets for Calculating Spin–spin Coupling Constants. *Theor. Chem. Acc.* **2010**, *126*, 371–382.

(S15) Sauer, S. P. A. Second-order polarization propagator approximation with coupled-cluster singles and doubles amplitudes – SOPPA(CCSD): the polarizability and hyperpolarizability of Li^-. *J. Phys. B* **1997**, *30*, 3773–3780.

(S16) Enevoldsen, T.; Oddershede, J.; Sauer, S. P. A. Correlated calculations of indirect nuclear spin-spin coupling constants using second-order polarization propagator approximations: SOPPA and SOPPA (CCSD). *Theor. Chim. Acta* **1998**, *100*, 275–284.

(S17) Kjær, H.; Sauer, S. P. A.; Kongsted, J. Benchmarking NMR indirect nuclear spin-spin coupling constants: SOPPA, SOPPA(CC2), and SOPPA(CCSD) versus CCSD. *J. Chem. Phys.* **2010**, *133*, 144106.

(S18) Oprea, C. I.; Rinkevicius, Z.; Vahtras, O.; Ågren, H.; Ruud, K. Density functional theory study of indirect nuclear spin-spin coupling constants with spin-orbit corrections. *J. Chem. Phys.* **2005**, *123*, 014101.

(S19) Møgelhøj, A.; Aidas, K.; Mikkelsen, K. V.; Sauer, S. P. A.; Kongsted, J. Prediction of spin-spin coupling constants in solution based on combined density functional theory/molecular mechanics. *J. Chem. Phys.* **2009**, *130*, 134508.

(S20) Khaliullin, R. Z.; Cobar, E. A.; Lochan, R. C.; Bell, A. T.; Head-Gordon, M. Unravelling the Origin of Intermolecular Interactions Using Absolutely Localized Molecular Orbitals. *J. Phys. Chem. A* **2007**, *111*, 8753–8765.

(S21) Khaliullin, R. Z.; Bell, A. T.; Head-Gordon, M. Analysis of Charge Transfer Effects in Molecular Complexes Based on Absolutely Localized Molecular Orbitals. *J. Chem. Phys.* **2008**, *128*, 184112.

(S22) Kühne, T. D.; Khaliullin, R. Z. Electronic Signature of the Instantaneous Asymmetry in the First Coordination Shell of Liquid Water. *Nat. Commun.* **2013**, *4*, 1450.

(S23) Kühne, T. D.; Khaliullin, R. Z. Nature of the Asymmetry in the Hydrogen-Bond Networks of Hexagonal Ice and Liquid Water. *J. Am. Chem. Soc.* **2014**, *136*, 3395–3399.

(S24) Elgabarty, H.; Khaliullin, R. Z.; Kühne, T. D. Covalency of Hydrogen Bonds in Liquid Water Can Be Probed by Proton Nuclear Magnetic Resonance Experiments. *Nat. Commun.* **2015**, *6*, 8318.
